# Do caffeine and alcohol affect fertility treatments?

**Published:** 2022-11

**OCTOBER 19, 2022** - A recent analysis published in Acta Obstetricia et Gynecologica Scandinavica found no association between women’s caffeine consumption and pregnancy or live birth rate after fertility treatments, but women’s alcohol consumption was associated with decreased pregnancy rate after treatments when weekly consumption was greater than 84 g (approximately 7 standard drinks).

Also, men’s alcohol consumption was associated with decreased live birth rate after fertility treatments in women when weekly consumption was greater than 84 g.

The analysis included all relevant studies published before July 15, 2022. A total of 7 studies on caffeine consumption and 9 studies on alcohol consumption were included, with a total of 26,922 women and/or their spouse who underwent fertility treatment.

Compared with abstainers, the chance of achieving a pregnancy after fertility treatment decreased by 7% for women who consumed 84 g alcohol per week, and the chance of partners achieving a live birth decreased by 9% for men who consumed 84 g alcohol per week.

“Couples should be aware that some modifiable lifestyle factors such as drinking habits may affect their fertility treatment outcomes. But how these factors impact the reproductive system still needs more research to elucidate,” said corresponding author Yufeng Li, MD, of Tongji Hospital, in China.

***URL upon publication: https://onlinelibrary.wiley.com/doi/10.1111/aogs.14464***

***Full citation:** “The association between caffeine and alcohol consumption and IVF/ICSI outcomes: A systematic review and dose–response meta-analysis.” Wentao Rao, Yuying Li, Nijie Li, Qingyun Yao,Y ufeng Li. Acta Obstet Gynecol Scand; Published Online: 19 October 2022 (DOI: 10.1111/aogs.14464).*

*Copyright © 2019 The Cochrane Collaboration. Published by John Wiley & Sons, Ltd., reproduced with permission.*

